# Supplementary figures and images for: Preliminary evaluations of 3-dimensional human skin models for their ability to facilitate in vitro the long-term development of the debilitating obligatory human parasite Onchocerca volvulus
Source: PLoS Negl Trop Dis. 2020 Nov 5;14(11):e0008503. doi: 10.1371/journal.pntd.0008503 (PMC7671495; doi:10.1371/journal.pntd.0008503)

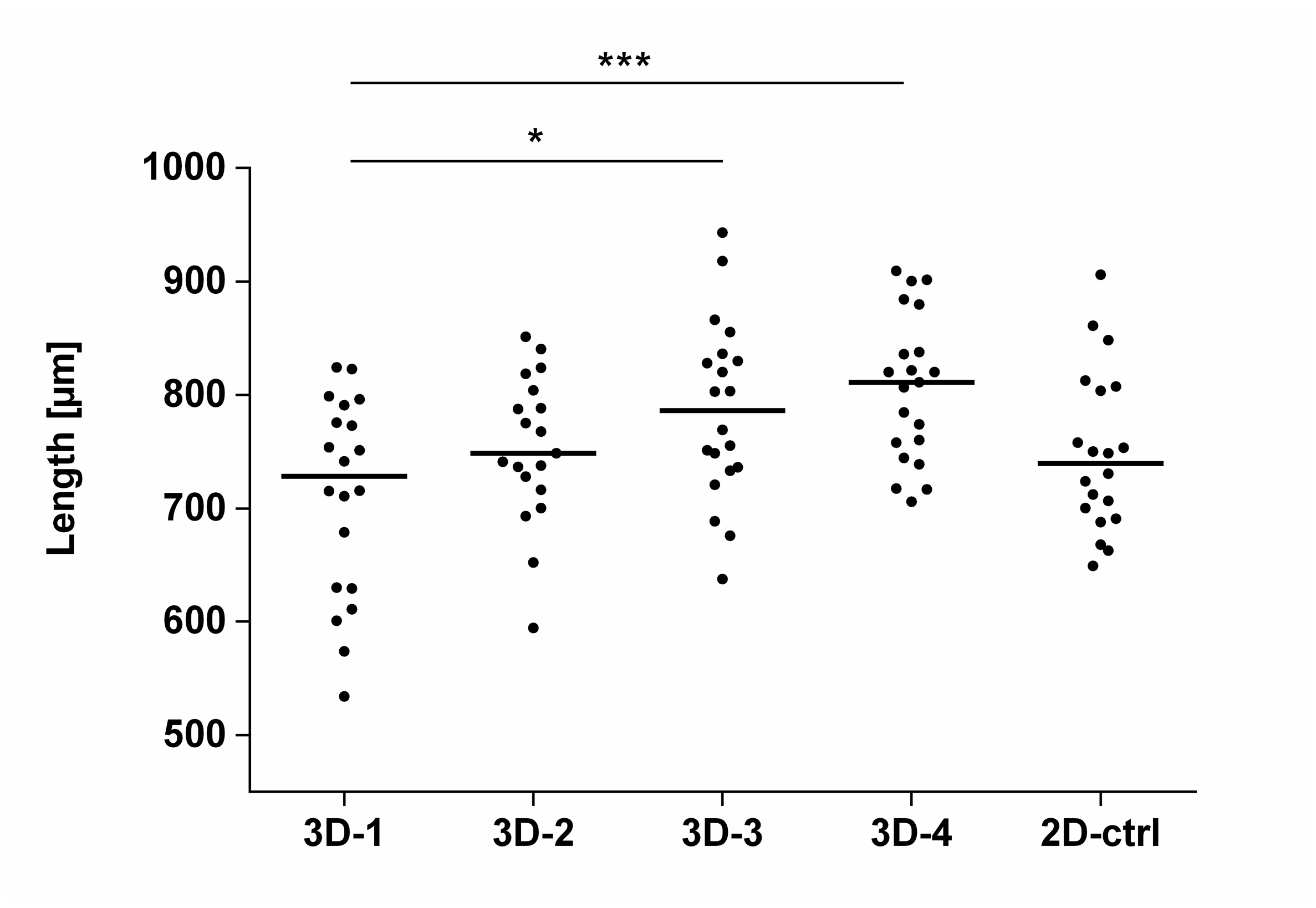

Supplement: S1 Fig — 3D-3 and 3D-4 demonstrated better growth than 3D-1 and 3D-2. (TIF) [file pntd.0008503.s001.tif]

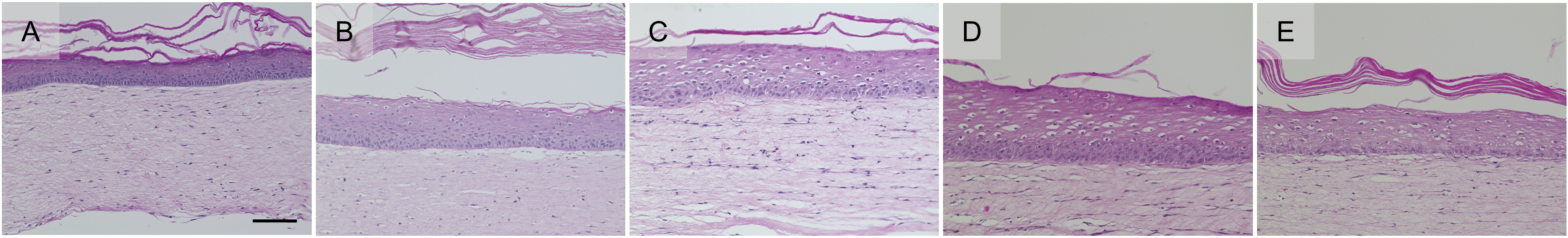

Supplement: S2 Fig — Hematoxylin and Eosin staining of FTSM cultured with experimental co-culture media. A. 3D-1 (E10), B. 3D-2, C. 3D-3, D. 3D-4. E. L4 medium. The tested media did not have a negative impact on dermal formation. All epidermis-specific layers stratum basale, spinosum, granulosum and corneum were formed. Compared to FTSM cultured with skin model specific E10 media, the epidermal tissues cultured with 3D-2 to 3D-4 and L4 medium shows changes in the cell layer formation, especially by vacuolization in the stratum spinosum. The lack of the stratum corneum can be explained by shearing off through histological processing. (TIF) [file pntd.0008503.s002.tif]

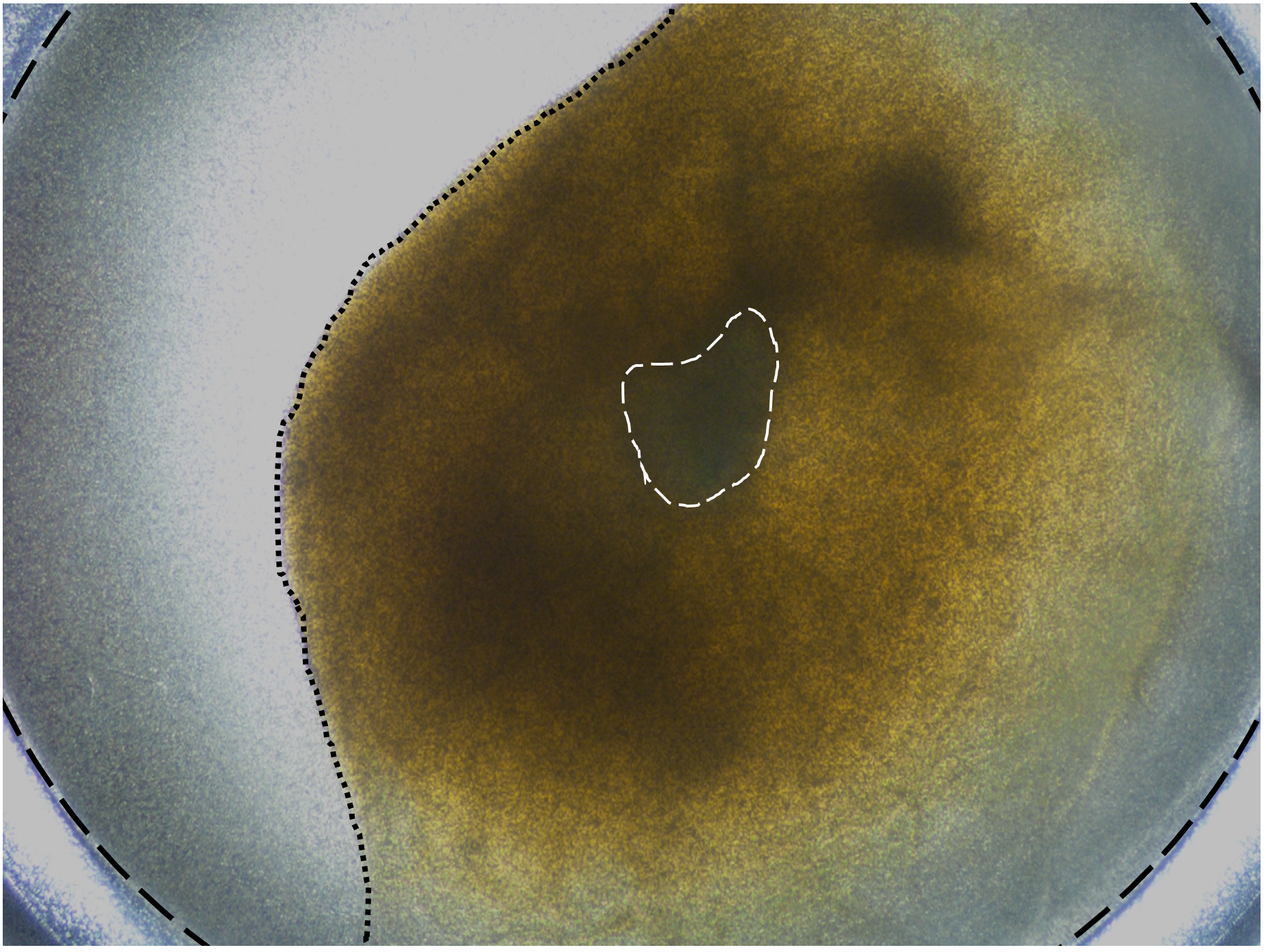

Supplement: S3 Fig — By day 35, due to the constant remodeling of the collagen by the hDF, the model contracted causing the imprinted compartment to collapse. The hydrogel loosened from the insert wall (dashed black line) and decreased in volume, shown by the spotted black line. The imprinted compartment containing the larvae shrank (dashed white line), impeding their observation. Magnification: 2X. (TIF) [file pntd.0008503.s003.tif]
