# Supplementary material for: Preliminary evaluations of 3-dimensional human skin models for their ability to facilitate in vitro the long-term development of the debilitating obligatory human parasite Onchocerca volvulus
Source: PLoS Negl Trop Dis. 2020 Nov 5;14(11):e0008503. doi: 10.1371/journal.pntd.0008503 (PMC7671495; doi:10.1371/journal.pntd.0008503)
Supplement: S2 Table — (DOCX) [file pntd.0008503.s005.docx]

| **Co-Culture set-up** | **Duration of co-culture**  **(worm age at end of culture)** | **Type of cell/ model used** | **Culture media**  **(composition detailed in S1 Table)** | **Contact with 3-D tissue models** | **Aim** | **Outcome** |
| --- | --- | --- | --- | --- | --- | --- |
| 2D -media testing  **(Fig. 3)** | 7 days  (21 days) | 2-D HUVEC monolayer | 3D-1  3D-2  3D-3  3D-4  L4 media (control) | N/A | Preliminary experiment to optimize media for an integrated culture of larvae and human skin models. | After a 7- test, **3D-3 [median length: 772.0 µm (range 649.5 – 858.1 µm)] and 3D-4 [median length: 785.2 µm (range 634.9 – 858.3 µm)]** were determined to be most suitable for long term culture of larvae. The 2D-control was at median length to **761.0 µm (range 680.1 – 841.0 µm).** |
| Direct co-culture set up- **(Fig. 5)** | 28 days  (43 days) | Compressed dermal models (no keratinocytes) |  | + | To test the effect of culturing larvae in direct contact with the worms. | Though worms grew significantly during the culture period [**786.4 µm (range 701.4 - 875.8 µm)]**, it became increasingly difficult to monitor the worms as they migrated away and possibly into the models.  After **28 days in culture, 44%** of the worms disappeared and could not be further monitored.  This system did not prove feasible for monitoring of worms in long term culture. The indirect co-culture was adapted to circumvent this. |
| Indirect co-culture setup  **(Fig. 6)** | 77 days  (92 days) | FTSM | 3D-3  3D-4 | - | To overcome issues of monitoring worms that were experienced during the culture period while culturing in contact with the models.  Worms maintained in a separate transwell from the skin models, still allowing the exchange of soluble factors between them. | Both 3-D conditions tested improved the median length of the worms with **3D-3 at 830.8 µm (range 765.1 - 897 µm) and 3D-4 at 816.3 µm (range 744.4 - 934.8 µm)** compared to **2-D control** which was at **766.6 µm (range 634.8 - 836.6 µm)** after 77 days in culture.  However, in terms of viability (the % of worms that remained motile from start to end of culture), the 3D-4 (30.7%) condition was 2-fold more viable than 3D-3 (15.8%) and comparable to 2D control (30%).  3D-4 media was determined to be the best combination for long term co-culture of worms and models allowing for significantly improved growth and comparable viability to the traditional 2D system. |
|  |  | Control 2-D HUVEC monolayer  (control) | L4 media  (2D-ctrl) | N/A |  |  |
| Direct and indirect co-culture with Adipose aggregates  **(Fig. 8)** | 35 days  (52 days) | Adipose aggregates | 3D-4 | + | To assess the effects on viability when worms were cultured with and without contact with the adipose aggregates.  The nature of this model allowed for monitoring of worms even when maintained in direct contact with the larvae. | This system demonstrated worms cultured in direct contact **(95%)** with the adipose aggregates had significantly improved motility over time compared to those grown in indirect contact **(37%)** with the adipose aggregates and 2D control **(26%)** conditions.  This experiment highlights the benefits to viability by culturing in contact with the adipose aggregates and warrants for further studies integrating these models with the FTSM system for long term cultures. |
|  |  |  |  | - |  |  |
|  |  | Control 2-D HUVEC monolayer | L4 media | N/A |  |  |

**S2 table:** A summary of the experiment goals and the outcomes of the various preliminary studies in which we optimized culturing condition that support the growth and fitness of *O. volvulus* larvae cultured *in vitro*.
