# Supplementary material for: Preliminary evaluations of 3-dimensional human skin models for their ability to facilitate in vitro the long-term development of the debilitating obligatory human parasite Onchocerca volvulus
Source: PLoS Negl Trop Dis. 2020 Nov 5;14(11):e0008503. doi: 10.1371/journal.pntd.0008503 (PMC7671495; doi:10.1371/journal.pntd.0008503)
Supplement: S1 Text — (DOCX) [file pntd.0008503.s009.docx]

**List of abbreviations included in the manuscript:**

2-dimensional (2-D)

3-(4,5-dimethylthiazol-2-yl)-2,5-diphenyltetrazolium bromide (MTT)

3-dimensional (3-D)

3-isobutyl-1-methylxanthine (IBMX)

Adipogenic differentiation medium (ADM)

Analysis of variance (ANOVA)

Dulbecco’s Modified Eagle’s Medium/Nutrient Mixture F-12 Ham (DMEM/F12)

Endothelial Cell Growth Medium (EGM-2)

Experimental co-culture media (3D-1 to -4)

Extracellular matrix (ECM)

Fetal bovine serum (FBS)

Full-thickness skin model (FTSM)

Hematoxylin and Eosin (HE)

Human epidermal keratinocytes (hEK)

Human dermal fibroblasts (hDF)

Human mesenchymal stromal cells (hMSC)

Human umbilical vein endothelial cells (HUVEC)

Iscove’s Modified Dulbecco’s Medium (IMDM)

Minimum essential medium alpha (MEM-alpha)

New York Blood Center (NYBC)

Optimized adipogenic differentiation medium (ADM+)

*O. volvulus* third-stage larvae (L3)

*O. volvulus* fourth-stage larvae (L4)

*O. volvulus* pre-adult stage (L5)

Paraformaldehyde (PFA)

Peripheral blood mononuclear cells (PBMCs)

Phosphate buffered saline (PBS)

Scanning electron microscopy (SEM)
