# Supplementary material for: Preliminary evaluations of 3-dimensional human skin models for their ability to facilitate in vitro the long-term development of the debilitating obligatory human parasite Onchocerca volvulus
Source: PLoS Negl Trop Dis. 2020 Nov 5;14(11):e0008503. doi: 10.1371/journal.pntd.0008503 (PMC7671495; doi:10.1371/journal.pntd.0008503)
Supplement: Attachment — Submitted filename: Response to reviewers.doc [file pntd.0008503.s010.doc]

To the Editorial Office – PLOS Neglected Tropical Diseases

|  | **Bitte bei Antwort angeben:** | |  |
| --- | --- | --- | --- |
| Ihre Nachricht vom: | Unser Zeichen: | Telefon: (09 31) 31 81209 | Würzburg, 11/30/2026 |
| Ihr Zeichen: |  | Telefax: (09 31) 31 81068 |  |
| Fallnummer: |  | E-Mail: Jan.Hansmann@uni-wuerzburg.de | |

**Optimizing 3-dimensional human skin models to facilitate long-term development of Onchocerca volvulus, a debilitating obligatory human parasite**

Leitung
Prof. Dr. Jürgen Groll

Sekretariat

Tel.: (0931) 31-80183

Fax: (0931) 31-81068

Dear Dr. Keke Fairfax,

We hereby would like to submit the revision of our manuscript entitled: “Optimizing 3-dimensional human skin models to facilitate long-term development of *Onchocerca volvulus*, a debilitating obligatory human parasite”. We thank you very much for your assistance and handling of our manuscript.

According to the reviewer’s suggestions, we have changed the title to:

**Preliminary evaluations of 3-dimensional human skin models for their ability to facilitate *in vitro* the long-term development of the debilitating obligatory human parasite *Onchocerca volvulus***

Our manuscript has been carefully revised and we respond to the comments and suggestions of the reviewers point by point (see below).

We thank the reviewers for the valuable comments and the effort to improve the manuscript. Furthermore, we are grateful for the encouraging words from the reviewers.

For revision, we highlighted all changes. Additionally, we prepared a plain version of the manuscript. All authors have approved this revised version.

Yours sincerely,

Jan Hansmann

**Point-to-point responses.**

**Reviewer #1:**

Malkmus et al. presented two 3-dimensional in vitro culture that allow long-term culturing of Onchocerca volvulus and facilitate development of L4 larvae to young adults. The results shown here are very interesting and crucial for the filarial research community, since a source of O. volvulus worms is still missing but is indispensable for research about the parasite’s biology and development of macrofilaricidal drugs. In my opinion, the manuscript is suitable for publication in PLOS NTD, but a couple of points need to be clarified beforehand.

**Response:**

We are very grateful for this feedback and the classification of our data as supportive for the filarial research community. We also appreciate the comments on how to improve the manuscript.

Major comments:
1) Did the authors try to directly use and implement the L3 larvae into the culture systems? This would allow a continuous observation within one culture system.

**Response:**

We would like to thank the reviewer for this very constructive suggestion of covering more of the life cycle of *O. volvulus, in vitro*. Nevertheless, in this study we were time-efficient because of the maximum duration of the FTSM culture, we opted to use L4 that were generated in 96 well plates in the presence of PMBCs. Thus, we could save one week of additional culture.

We already started to experiment with the indirect culture setup (two inserts inside one culture well – one insert with larvae and one insert with tissue) and will definably test whether adding the L3s into this setup will increase fitness of the L4s and pre-adult with time. Moreover, we found in these preliminary experiments that a beneficial effect on development could be anticipated because the efficiency of molting increased from 26.8 % in the 2D culture to 40 % in the presence of the FTSM. However, these preliminary results need to be further elucidated.

For clarification, we adapted and added the following sentences to the discussion in line 731 – 738.

“For example, we could use a simplified and yet advanced experimental designs to establish the individual impact each of the tissue components (e.g. adipose tissue, dermis, and epidermis, varied but defined immune cells) have on larval fitness and development through the two molts that happen in the human host. We could possibly start the *in vitro* varied culturing conditions by initiating the culturing with the L3s and compare to the development of the L4s versus when the complexed co-culturing starts with the L4s; such conditions could resemble more of the parasite’s lifecycle.”

2) In general, why did the authors stop the cultures after 77days (collagen-based tissue model) and 52 days (adipose tissue model), respectively? Did the motility/survival reduce after these time-points? Interestingly, Figure 8D showed that the motility in the direct adipose tissue model remain high and constant upon 52 days compared to the start of the culture.

**Response:**

In this study we aimed to improve larvae fitness in long-term culture. As shown in Fig. 1, *O. volvulus* larvae undergo a massive drop of viability [%] until 7 weeks of culture in the 2D system with a HUVEC feeder layer. We aimed to achieve improved growth of larvae to the preadult stage. Based on prior experience from 2D, L4 larvae undergo a molt to the preadult stage around day 65 in culture. We hence maintained the worms in culture D77 in anticipation of finding an improved growth in comparison to 2D conditions. We ended the indirect co-cultures as the viability of this specific batch of worms was much reduced past this timepoint.

In the adipose tissue co-cultures, we demonstrated that larvae in direct contact to adipocytes could be maintained without the significant loss of viability even when co-cultured for 52 days, roughly 7 weeks. Future studies will be planned to compare timelines as well as to expand periods to prove the long-term effect of 3D skin tissue models on larvae fitness *in vitro*.

The study presented in the manuscript was a comparative proof of concept study that allowed us to identify the best co-culturing condition first. We therefore now also adapted the title to highlight the premise of this study to “**Preliminary evaluations of 3-dimensional human skin models for their ability to facilitate *in vitro* the long-term development of the debilitating obligatory human parasite *Onchocerca volvulus***”.

To clarify this aspect, we changed the following sentences to the introduction and the discussion.

**Introduction:** Line 166 – 169: “In this exploratory proof of concept study, we examined the influence of three human subcutaneous tissue components (Factors 1 – 3) on the ability to improve the fitness of long-term cultured *O. volvulus* larvae *in vitro* as compared to the traditional 2-D culture system. “

**Discussion:**

Line 579: “Viability drops in the first 7 weeks very rapidly.”

Line 582 – 583: “The study focused on the first 7 weeks, when usually the drop of viability is detected.”

Line 717 – 723: “We hypothesize that by increasing the complexity of the *in vitro* 3-D culturing system will not only achieve a synergetic effect of the FTSM (growth) and the adipose tissue (viability) on larval fitness, but also when combined with direct co-culturing conditions that support monitoring over a prolonged culture period would also potentially enhance further the survival and development of L4 to the pre-adult juvenile worms (as already done) and hopefully also to the more mature adult worms.”

We hope that we were able to address the comment sufficiently.

3) In regards to the question in point 2), can the authors show the motility and survival rates for Figure 6D and length and survival rate for Figure 8D?

**Response:**

As mentioned in the revised introduction, the aim of the study was to test the impact of the enhanced culture conditions on the growth and viability of the worms. The growth of the larvae is definitely a very important parameter. However, in the chosen timeframe for proving the enhanced viability where typically the loss of motility is detected, we did not anticipate a significant growth. The major increase in length occurs around day 70 of worm age during molting to L5. Considering figure 8D, the growth of the larvae in this timeframe of 52 days was not a relevant parameter and was not significantly changed. Now, that we know that this culture setup has a supporting effect on larvae survival, future experiments will also focus not only on the growth but also other parameters. We added the following to the manuscript in line 717 – 723.

“We hypothesize that by increasing the complexity of the *in vitro* 3-D culturing system will not only achieve a synergetic effect of the FTSM (growth) and the adipose tissue (viability) on larval fitness, but also when combined with direct co-culturing conditions that support monitoring over a prolonged culture period would also potentially enhance further the survival and development of L4 to the pre-adult juvenile worms (as already done) and hopefully also to the more mature adult worms.”

Considering Figure 6D, here the length of motile worms only is presented in the graph. Unfortunately, a one to one assignment is not possible as we cannot label individual worms. Nevertheless, the % of motile worms was recorded for this experiment at ~35 % for all three groups studied at day 70 of culture and dropped to 30 % for 2-D condition and a comparable 30.7 % in the 3D-4 group on day 77. The 3D-3 group was not as supportive of viability toward the end of the culture period (15.8 %).

We would like to refer to line 486 – 492 in the results section of the manuscript:

“However, the 3D-3 media was not as supportive as 3D-4 in maintaining the motility of the developing larvae; 2-fold more larvae were motile in 3D-4 on day 77 of culture. Interestingly, a new observation was made; fibroblasts from the FTSM migrated towards the culture insert containing the larvae (**Fig. 6A**), which resulted in the integration of the L4 into a newly formed cluster of hDF and ECM within their own insert (**Fig. 6C**). This has been observed as early as day 21 of culture and without affecting too much the fitness of the larvae over time.”

To further clarify this point, we added the % of motile larvae to figure 6D. We hope that these edits have addressed the reviewer’s comment sufficiently.

4) Since the authors state that fibroblasts from the FTSM migrate towards the insert containing the larvae leading to cluster formation, I am wondering if fibroblasts alone could be used for good culturing condition without the whole FTSM. Indeed, the authors discussed that tissue components need to be tested separately and questioned the presence of the epidermal layer for O. volvulus development. In addition, the connection between fibroblasts and nodule formation have been already shown, it would be really interesting and important for the message/conclusion of the manuscript, if the authors could state/discuss preliminary results or hinds that fibroblasts alone might be beneficial for O. volvulus development and maybe also nodule formation.

**Response:**

We acknowledge the reviewer’s comment on fibroblast impact. It has already been demonstrated that hDF have a beneficial effect on survival/growth in the 2D culture setup used as a feeder layer and thus proving the soluble factor exchange (Voronin et al., 2019). In the study presented here, we show that the exchange of soluble factors drives the recruitment of fibroblasts towards the larvae. This is what we wanted to highlight in the discussion in line 637 – 642 of the manuscript.

“However, in this setup, the insert containing the larvae was placed together with the second insert containing the FTSM within the same tissue culture well **(Figs. 6A-B)**, thereby still enabling the bi-directional communication via soluble factors. Interestingly, this indirect co-culture with L4 resulted in a strong migration of hDF from the tissue model towards the larvae and the formation of ECM, which possibly confirms active crosstalk between the larvae and the tissue model.”

We are very grateful for the reviewer’s recommendation to further strengthen this finding.

In contrast to the adipose tissue models that only supported the larval fitness in the direct contact setup, the hDFs were acting effectively in both conditions (indirect/soluble and direct contact). This fact seems to be important regarding nodule formation and will be addressed in future studies.

We would like to refer to the following information in the discussion:

Line 649 – 653:

“We wonder if the recruitment of cells from the skin tissue model towards the larvae could possibly be an initial step of triggering nodule formation by the *O. volvulus* worms. Like the nodular niche, *O. volvulus* larvae were seen to be integrated into the formed cluster of cells and ECM secreted by the hDF, as demonstrated by the SEM **(Fig. 6C)**.”

We also added the following sentences to the discussion in line 660 – 662:

“This finding is a hint that fibroblasts have a crucial role in the culture of the larvae and can strongly interact with the parasite even without an immune system that usually modulates the hosts’ response.”

However, to further study the interaction of incorporated *O. volvulus* within the 3-D hDF seeded collagen scaffolds, advanced microscopy methods need to be applied to enable monitoring of larval fitness *in vitro*.

We would like to refer to line 682 – 686 of the manuscript.

“In contrast to the direct culture in the skin models, the adipose tissue models consisting of cell aggregates placed on the permeable membrane of the culture insert allowed the visual observation of the L4 cultured in a direct contact with the adipocytes by light microscopy **(Fig. 8C)**. “

To further highlight this aspect, we added the following to the discussion in line 666 – 672.

“To further study whether this interaction is beneficial, we will need to overcome the challenges we had with the direct contact co-cultures. Particularly, advanced microscopy methods need to be established and applied to enable non-invasive monitoring of larval fitness during the long-term cultures versus determining the vitality and growth of larvae using assays that are irreversible such as the digestion of the collagen scaffold followed by MTT (3-(4,5-dimethylthiazol-2-yl)-2,5-diphenyltetrazolium bromide) assay.”

We hope that we could respond satisfactorily.

5) Since the parasites did not get in contact with the tissue/cells (except the fibroblasts) from the FTSM, did the authors measured soluble factors/molecules (cytokines, chemokines, growth factors etc.), which might be secreted from the FTSM (and also adipose tissue)?

**Response:**

No, unfortunately we did not yet test the soluble factors that might have contributed to the migration or improved fitness on a molecular level. The focus of this study was mostly about finding the best conditions for the cultures so we can in future studies focus on the mechanisms. Now, as we know about the supportive effect of FTSM and adipose cells on L4 larvae fitness, we will analyze the soluble components in the supernatants of the next generation of co-culture experiments.

We added the following to the discussion in line 743 – 749.

“Also, it would be of interest to analyze and compare the secreted from the FTSM, adipose tissue and/or combined tissues when cultured with live L4s versus killed L4s to validate if the soluble factors/molecules (i.e. cytokines, chemokines, growth factors etc.) are different and thus in response to the presence of actively growing larvae. Our human *in vitro* tissue system, therefore, can enable novel opportunities to answer host-parasite interaction questions that cannot otherwise be studied in the human host or in the existing animal models.”

6) In general, the authors should compare and state/discuss what tissue model is preferable for the development of O. volvulus and should be used for further research.

**Response:**

In this study we present the proof of concept that *O. volvulus* L4 larvae can be co-cultured with different in *vitro tissue* models. So far, we have demonstrated a benefit of the direct interaction between cells and larvae, as well as soluble factors secreted by the FTSM/hDF are possibly also supporting larval fitness.

In our opinion, the two most promising co-culture approaches for future studies are represented by the indirect FTSM co-culture based on soluble factor exchange, improving the **growth of larvae**. As already discussed in the manuscript in lines 727 – 731 it could be applied in futures studies of parasite-host interaction and communication.

“Although we did not initially consider in this proof of concept studies the indirect co-culture setup as an optimal culture condition for *O. volvulus*, we discovered that this culture system could be still a valuable method to unraveling the molecular basis that support the formation of the nodule and the interactions between the parasite and the distinct human host tissues within the nodule.”

According to point 5, this will be combined with the analysis of the soluble factors exchanged through the changed composition of the culture supernatant. The improved motility in the co-culture of adipose aggregates also represents a promising system, which needs to be further studied before it can be applied to the long-term cultures and to better understand the molecular mechanisms that will support our ultimate goal of growing adult worms in a dish.

These results suggest that the integration of both tissue models in one system may synergize their individual contributions to the enhanced of motility and growth. Therefore, our most promising next approach will be to culture the larvae within the adipose tissue (improved motility) together in the same culture well with an extra insert containing the FTSM (improved growth). After setting up the monitoring methods (mentioned in the response to comment 4), also direct culture in a FTSM with an integrated adipose tissue could be performed.

Like in the response of comment 2 we added the following to the manuscript in lines 717 – 723.

“We hypothesize that by increasing the complexity of the *in vitro* 3-D culturing system will not only achieve a synergetic effect of the FTSM (growth) and the adipose tissue (viability) on larval fitness, but also when combined with direct co-culturing conditions that support monitoring over a prolonged culture period would also potentially enhance further the survival and development of L4 to the pre-adult juvenile worms (as already done) and hopefully also to the more mature adult worms.”

We hope that we were able to clarify accordingly.

Minor comments:
1) Lines 196 and 275: O. volvulus in italics

🡪 fixed
2) Scale bar is missing in Figure 4D and E

🡪 fixed
3) Line 554: in vitro in italics

🡪 fixed

We thank the reviewer for the thorough evaluation of the manuscript and the constructive comments.

**Reviewer #2:**

Comments to the authors

This manuscript reports on the development of an in-vitro 3-D tissue model to achieve in long-term cultures finally mature adult Onchocerca filariae for drug development and biomedical research. The authors aimed to provide a valuable source also of worms at various developmental stages. This investigation represents a highly relevant and promising innovative goal, but also a challenging ambition. The submitted data represent encouraging first results which have to be extended and further developed. The study and the sequence of experiments are largely scientifically sound, the results mostly clearly presented, figured in illustrations and photographs. Overall, this manuscript is relevant for future biomedical research on filariae and their control by novel drugs and should be published after revision to address all specified comments and concerns.

Notably, the ambitious claims are highly demanding but should be to a degree toned. Thus, the title states „Optimizing 3-dimensional human skin models ...“ that interested helminthologists will assume that 3-D skin models have been already applied as source of developed nematode stages and in this report are further optimized. The title could read „First development of a 3-dimensional human skin models to facilitate long-term development of Onchocerca volvulus, a debilitating obligatory human parasite“. The authors claim to (i) establish a source of adult Onchocerca filariae, (ii) to contribute to the progress of anti-parasitic treatment strategies for various helminth infections, (iii) to support the development of new interventional tools to fight this debilitating obligatory human parasite - represent well-aimed future goals, (iv) to reveal a proof of concept - but the presented data constitute here first but very relevant and successful results for this aim. The intended long-term 3-D FTSM culture over multiple weeks at least appears very demanding and complex and further has to be elaborated and optimized.

The experiments widely comprise and compare 2-D culture conditions with 3-D tissue models exploring numerous media and larvae. The denotation, however, of the multiple applied media and their allocation to the numerous 2-D/3-D culture conditions appear considerably complex and rather transparent and should be improved. Further, the multitude of abbreviations has to be explained in a list.

**Response:**

First, we would like to thank the reviewer for the positive feedback and consideration of our manuscript for publication. Second, we appreciate the very sound comments on the placement of our research goals and the already achieved progress that we present in our manuscript. The comments help us to understand that the data we show are just a very early step to achieve the aims claimed in i-iii.

We adapted partially the title as recommended by reviewer #2 and we agree that the revised title emphasizes the proof of concept character of the study and highlights the novelty for the *O. volvulus* research community in particular. The new title was adapted as followed: **“Preliminary evaluations of 3-dimensional human skin models for their ability to facilitate *in vitro* the long-term development of the debilitating obligatory human parasite *Onchocerca volvulus*”**

To support the readability of the manuscript, we prepared a list of all abbreviations. If the article is accepted, we will make sure that during the processing of the article the list of abbreviations will be included.

Major points

The formation of an onchocercoma in the subcutaneous tissue of an infected host represents a host reaction to encapsulate the worm parasite and to prevent its further migration and development but foster its degeneration (PMID: 14738900, PMID: 1401100). Thus, the migration of adipocytes, mesenchymal stromal cell and dermal fibroblasts to the worm larvae and adherence of such host cells to the foreign surface are in agreement with the formation of the nodule. However, the accentuation that co-culturing of worm stages benefits to larval motility and fitness by assumed secreted factors as a positive result of the model - rather may reflect the host activity to constrain and cope with the invading parasite. Further, the complex full-thick skin model comprising epidermal keratinocytes, fibroblasts, stromal cells and adiposic cell types can admit the survival and development of L4 to young and subsequent mature adults – but this observation does not match the biological role of nodule formation which are one general goal of these experiments.  In the infected host the worm stages can develop within in emerging onchocercoma rather by factors secreted by the parasite which hinder its restriction and damage – than by compounds secreted by host cells to foster and promote the parasites fitness and development, as suggested. The authors should reconsider the acting biological principles. There exists no biological regularity of the host to foster the fitness and development of its parasite. The authors should not mix and permute the natural biological processes and the target of these experiments namely to receive Onchocerca stages for (i) analysis of the parasite general biology (morphology, transcriptome and proteome profiling to validate their stage of development) as well as (ii) for immunological and vaccine experiments and (iii) for treatment trials.

The authors stated no detrimental effects on larvae by the applied stromal cells in the proximity of adipose aggregates, they have, however, to consider that in vivo onchocercoma innate and adaptive immune cells of the host can damage the worm stages by secreted toxic proteins and radicals (PMID: 2014134, PMID: 1980799, PMID: 6879705).

**Response:**

Immune cells play a significant role in the creation and characteristics of the natural niche of *O. volvulus* in infected individuals, the onchocercoma. In future studies, it would be important to also integrate defined immune cells to our tissue model to further reproduce the various components within the habitat of developing worms and thus the more genuine outcomes of the host-parasite interactions that are at play. Unfortunately, our proof of concept studies has not yet produced the proper conditions that would allow exploring also the function of immune cells within the 3D skin model we have examined. Furthermore, for optimal cross talk investigation between all cell types and tissue components, especially for the use of immune cells within a model system, the cells need to be derived from a single donor.

The reviewer is right that the onchocercoma is driven by the response of the host to the parasites and we believe based on the literature (we added many relevant new citation) that it is also triggered by molecules secreted by the parasite. It takes more than 10 years for the parasites to die within the onchocercoma and during that period the parasite continues to grow, mature and become reproductive and secrete microfilariae. Therefore, it is two-way beneficial interactions, the parasites get products from the host that it needs for its fitness while the host confines the worms so they won’t harm the host. Our preliminary studies show that cells can migrate to the transwell where the larvae resided probably triggered by factors that are secreted by the larvae while it also supports their fitness, thus the two-way beneficial interactions. Future studies will have to prove this, and we predict that once we have optimized the 3D models, we could study further the host-parasite interactions.

Thus, we decided to reduce the complexity of the adipose tissue to adipocytes and its progenitors that can be found within the aggregate model.

We added the following in the discussion, Lines 731 – 740: “For example, we could use a simplified and yet advanced experimental designs to establish the individual impact each of the tissue components (e.g. adipose tissue, dermis, and epidermis, varied but defined immune cells) have on larval fitness and development through the two molts that happen in the human host. We could possibly start the *in vitro* varied culturing conditions by initiating the culturing with the L3s and compare to the development of the L4s versus when the complexed co-culturing starts with the L4s; such conditions could resemble more of the parasite’s lifecycle. Furthermore, this could be easily combined the culturing system with other supplementary factors to reflect host responses to the parasite infection on a molecular level [6].”

I hope we could address the reviewer’s comment adequately.

Critical is the formulation „L5, early pre-adult stage“ since according to the filariasis scientific community the developmental stages of all filariae are microfilaria – L1 – L2 – L3 – L4 – juvenile adult – mature adult. There are rather no significant publications on L5-early pre-adult stages but Voronin, 2019 (PMID: 30653499) and formerly Duke, 1991 (PMID: 1888206). A myriad of scientists of classical parasitology on filariasis had studied predominantly 20-50 years ago the developmental stages of Onchocerca as of other filariae (PMID: 9754295, PMC305371, PMID: 2794455, PMID: 20976099, PMID: 27881553, ...). The report from Duke (PMID: 1888206) could not be verified. In the recent publication molecular, immunological or cellular parasitologists (PMID: 30653499) did not reference the existence of pre-adult fifth-stage larvae. Detached cuticles have to be doubtless verified from both, L3 and subsequently from the generated L4. Of interest, the author Sarah Lustigman addressed L4 and next adult worms in a previous publication (PMID: 1582477).

**Response:**

We agree with the reviewers that in *O. volvulus* clinical research parasites after they became fourth-stage larvae are regarded as juvenile adult because rarely one could have recovered such developmental stages of the parasites from collagenase treated nodules excised form infected individuals. Therefore, nobody could have been able to refer to the classical stages of nematodes beyond L4. However, based on parasitic nematode biology in most cases infection starts with third stage-larvae, L3 and they have to molt twice to L4 and then to L5 before they can be called adult. In our previous study in PLOS NTD (PMID: [30653499](https://www.ncbi.nlm.nih.gov/pubmed/30653499)), we called them L5 once we have observed molting based on a clear separation of cuticles during their molting process from L4 to L5 on days 48 – 60 while molting process was completed by days 60 – 75 which is accompanied by the appearance of a unique ultrastructure on the surface of the cuticle of these worms. We also called these L5 stage larvae pre-adult and provided molecular markers to verify that the OvL5s are pre-adult worms; these stages of the parasite will start growing without any additional molt to become mature and reproductive adult female and male worms. Our goal is to grow *in vitro* more of such fit pre-adult worms that can mature to reproductive parasites; the path to such an achievement is still long but is more achievable now once we have uncovered factors needed for their improved fitness *in vitro*.

We revised the manuscript and used the correct formulation in several places. Thank you for highlighting this issue.

Minor comments

As vitality and fitness criteria also the MTT assay can be applied based on the conversion of water soluble MTT (3-(4,5-dimethylthiazol-2-yl)-2,5-diphenyltetrazolium bromide) to an insoluble formazan product (PMID: 32628671).

**Response:**

We agree on the MTT assay to confirm the vitality of larvae additionally to the scoring based on movement. Due to the endpoint analysis of MTT we did not apply the assay to confirm viability during the ongoing culture. We have selected to base it on motility and appearance of the worms. Thank you very much for the advice. Furthermore, the following information can now be found in the manuscript in line 667 – 672:

“Particularly, advanced microscopy methods need to be established and applied to enable non-invasive monitoring of larval fitness during the long-term cultures versus determining the vitality and growth of larvae using assays that are irreversible such as the digestion of the collagen scaffold followed by MTT (3-(4,5-dimethylthiazol-2-yl)-2,5-diphenyltetrazolium bromide) assay.”

Reference 2 should be appropriately PMID: 31723729.

**Response:**

We changed the reference 2 according to the reviewer’s suggestion.

Line 275: O. volvulus in italics

🡪 fixed

We thank the reviewer for the thorough evaluation of the manuscript and the constructive comments.

**Reviewer #3:**

Comments to the authors

This manuscript reports progress on attempts to improve culture conditions for the development of Onchocerca volvulus larvae. There is a need for macrofilaricides (adulticides) and alternative approaches to ensure the eventual elminiation of river blindness. Adult parasites are difficult to source and culture, hampering efforts to directly study and screen this important life stage. No suitable small animal models support complete intra-mammalian development. This area of work is therefore highly significant, as it seeks to generate viable adult parasites from more readily sourced infective-stage larvae by optimizing in vitro culture approaches.

Previous work by the authors reported a 2D culture system that supported L4 to pre-adult (L5) development using feeder endothelial cells (HUVEC). Here, the authors explore the use of a hydrogel as a supporting matrix for 3D culture, co-culture with cell lineages matched to parasite niche, and direct contact between parasites and mammalian cells. Studies comparing four media conditions suitable for skin models, prioritized two media formulations (3D-3 and 3D-4) that led to better larval growth and motility. O. volvulus larvae were cultured with direct exposure to ECM and cells in a collagen-based skin model using a 'stamp' (mold) that allowed for some tracking of larvae. Indirect co-culturing was pursued to allow for better tracking, and also to allow the flow of cells and their secretions.

These experiments using the skin model are then supplanted by the adipose tissue model. Direct co-culture with adipose cells led to much more motile worms over a long time-course. The major limitations in this study are that the original goal of culturing viable adult stage worms has not been achieved, the primary molecular determinants of the improvements made are unclear, and the broad adoption of this protocol is made difficult by the requirement of primary cells. That said, these limitations are thoroughly discussed, and this work provides a great deal of new data that moves this field forward. This work may also guide approaches to culture other filarial or skin-associated nematode parasites.

**Response:**

We highly appreciate the opinion of reviewer #3 on the aim of our study. We agree on the limitations that come with our culture setups and that the original aim to produce adult stage worms was not achieved. However, we share the view of reviewer #3 that our culture approaches can be further developed and improved and hopefully contribute to the filarial research community, including supporting research of other skin related parasites.

We agree that our initial aims of producing adult *O. volvulus* worms were not achieved in this study. To highlight the explorative proof of concept character of this study we adapted to the title to **“Preliminary evaluations of 3-dimensional human skin models for their ability to facilitate *in vitro* the long-term development of the debilitating obligatory human parasite *Onchocerca volvulus*”**. We emphasize that our presented results of novel 3D culture approaches will hopefully support future studies that can meet the ultimate goal of culturing viable adult stage worms.

Moreover, we would like to highlight that our study indicated that the direct culture with adipose tissue increases motility/viability and indirect culture with the FTSM supports growth. Thus, a combination of these effects might be a strategy to achieve the initial goal. Therefore, our most promising next approach will be to culture the larvae within the adipose tissue (improved motility) together in the same culture well with an extra insert containing the FTSM (improved growth). After setting up the monitoring methods, also direct culture in a FTSM with an integrated adipose tissue could be aimed for.

We added the following to the manuscript in lines 717 – 723: “We hypothesize that by increasing the complexity of the *in vitro* 3-D culturing system will not only achieve a synergetic effect of the FTSM (growth) and the adipose tissue (viability) on larval fitness, but also when combined with direct co-culturing conditions that support monitoring over a prolonged culture period would also potentially enhance further the survival and development of L4 to the pre-adult juvenile worms (as already done) and hopefully also to the more mature adult worms.”

Reviewer #3 Responses to Questions:

**Methods**

Reviewer #3:

- Clearer description of what defines biological reps would be helpful throughout. It's unclear how parasites are sourced, cryopreserved, and thawed, with respect to potential batch effects. #

**Response:**

Hundreds of infected *Simulium sp.* are dissected 7-8 days post infection and the L3s collected from these flies, pooled, and then cryopreserved, each batch is recorded based on the date and number of larvae cryopreserved per tube. Each experiment was performed generally by combining few batches to generate the required numbers of L4s for the various experiments; thus, the L4 larvae in each experiment and between experiments are from multiple biological replicas. Regardless, we always cultured a small subset of the L4 using the 2D conditions to make sure that they are behaving similarly in these conditions across all experiments and therefore any differences in the 3D culture outcomes are not due to differences in L4 batches. Regardless, each culture condition had at least one technical replica.

The following information can now be found in the methods in line 276 – 289:

**“Preparation of *O. volvulus* larvae L4:** Various batches of cryopreserved L3s were thawed and washed with L3 wash media (described above). Each batch of L3s consists of pooled larvae that were recovered on the same day from hundreds of infected back flies and cryopreserved at the end of day. Each experiment was performed using 2 - 4 distinct batches of thawed L3s selected based on the expected average percentage molting and the number of L4s needed for the experiment. Accordingly, the L4s in each experiment and between the experiments listed below are from multiple biological replicas. Accordingly, L3s (10 per well) were cultured in 96-well-plates using the wash media that was supplemented with 20 % FBS in the presence of 1.5 x 10^5^ PBMCs per well. The cultures were maintained at 37 °C with 5 % CO2 for 7 - 14 days after which molting from L3 to L4 stages was determined. The L4 larvae were identified by their characteristic coiling movement in addition to the presence of the shed L3 cuticle. These worms were then collected using a stereoscope and used for further culturing in the various 3-D models and in 2-D culturing system as the control.”

- It's unclear in some places (e.g., Figure 8D) how many worms are being grouped in the summary data and whether these are technical replicates from the same batch or biological replicates from different thawing and molting events. In recognition of the difficulties in phasing experiments with 'true' biological replicates, I am only suggesting that this information be included - not necessarily suggesting added replication.

**Response:**

As explained above, each experiment was done with L4 that are composed by nature of multiple biological replicates as they come from different flies and different days of collections. Please see also the information that we added to the manuscript according to the previous comment.

However, for example, the data in Figure 2 are from at 18 batches of larvae, with each experiment performed with 300 to 600 larvae. To further clarify the use of biological replicates in each experiment, we added further information on numbers of larvae per well/insert to the figure legends of worm culture graphs. Thank you very much for this comment.

**Results**

Reviewer #3: - Figure 1 and 2 can be combined.

**Response:**

We have decided to separate the content in Fig. 1 and Fig. 2, as we would like to: (i) show the hypothesis of how one can translate from the *in vivo* niche of these worms into what can be done in an *in vitro* system. This is presented in the introduction, and (ii) present results of the 2D HUVEC culture system regarding the survival (not yet published data), which has been the basis why we took on the experimental challenge of culturing larvae with more complex 3D tissue models. We hope that the reviewer agrees to this argumentation.

- Figure 2: how many worms per well?

**Response:**

The results presented in figure 2 show a summary of the survival of 18 batches of larvae in the 2D cultures. Typically, 10 larvae are cultured within each culture insert above the HUVEC feeder layer. To clarify this aspect, and as discussed in the responses to the methodological comments, we added information regarding the use of multiple biological replicates in M&M and in the figure legends.

- Figure 4 would be helped by internal labels.

**Response:**

We appreciate these constructive comments to increase the legibility and comprehensiveness of our manuscript and adapted internal labels to Figs. 4A-C.

- Figure 5G: the d7 worm population includes at least one worm smaller than any initially measured (d0). Is this a dying worm, or does it reflect the difficulty in accurately measuring worm lengths?

**Response:**

Yes, a shrinking worm typically represents a dying one. However, with the direct contact system (fig. 5), sometimes, the worms were found in 3D conformations that were hard to measure accurately. This was likely the cause of seemingly “reduction“ in length in one of the worms within the pool. If removed as an outlier, statistically, it did not change the outcomes.

- L442: related to point above, could disappearing worms be dying, and not just moving through the scaffold?

**Response:**

We thank the reviewer for the comment. We would like to refer to the issues in larvae monitoring as described in the response to the previous comment. To clarify this aspect, the following revised information can be found in line 450 – 456:

“Unfortunately, due to the continuous remodeling of the collagen scaffold by the hDF the compartment collapsed (**S3 Fig.**), resulting in the disappearance of a significant number of the worms (reduction of 44 %) within the scaffold (**Fig. 5G**), which impeded the L4 monitoring and hence the long-term culturing of the larvae in direct contact with the skin models. Moreover, in the 3D culture system, the larvae are present in multiple layers, which makes it also more difficult to monitor their length accurately.”

- There are two Figure 7s uploaded

**Response:**

Thank you very much for highlighting this issue. We fixed it.

- It would be interesting to speculate (and eventually know) if much of the difference between direct and indirect co-culture motility in the adipose tissue model are a result of cholesterol / lipid availability, perhaps even from dying cells.

**Response:**

It is well known that worms need cholesterol (PMID: 12894170). In the adipocyte culture is a release of lipids, possibly also from dying cells. As the importance of lipids for developing worms is known, the standard 2D culture medium L4 is already supplemented with a defined lipid mixture. As discussed in Fig. 7 the lipid mixture did also support the adipogenesis, resulting in the faster accumulation of lipid vacuoles in developing adipocytes. We can see that the supernatant of the adipocyte aggregate cultures is enriched with lipids and thus appearing to be viscous as shown in a supplementary video we additionally added to the presented data (additional S3 video was uploaded). Moreover, this culture setup enables the direct contact of larvae and tissue/cells, which could be shown to be beneficial to larvae viability.

We added this aspect to the discussion in line 695 – 698: “The possible increased availability of lipids, fatty acids and cholesterol secreted by the adipocytes and/or released by the dead cells **(S3 Video)** contributing to the fitness of the developing larvae when they are cultured directly versus indirectly will need more studies.”

- A single summary table of all results (culture conditions, timelines, and mean lengths or normalized motility) would be helpful, even as a supplemental table.

**Response:**

We included a table of all results to the supplementary data. Thank you for the suggestion.

**Conclusions**

- A shorter version of much of the conclusion is warranted, given modest success in some areas and the supplanting of one approach with the next throughout. Most attention should be given to the culture approach that shows greatest promise (direct co-culture with adipose cells).

**Response:**

As we already mentioned, and it was emphasized by the comments to the authors, the results presented in this study are more of a proof of concept. Thus, we aimed to present all relevant data we collected so far.

In our opinion, the two most promising co-culture approaches for future studies are represented by the indirect FTSM co-culture based on soluble factor exchange, improving the growth of larvae. As already discussed in the manuscript, it could be applied in futures studies of parasite-host interaction and communication. To elucidate the molecular mechanisms these culture experiments will be analyzed for the soluble factors exchanged via the supernatant. The improved survival by the co-culture of adipose aggregates also represents a promising system, which needs to be further studied to be applied to the long-term cultures and to better understand the molecular mechanisms that support our ultimate goal of growing adult worms in a dish.

These results indicate that the integration of both tissue models in one system may combine their individual enhancements of viability and growth. Therefore, our most promising next approach will be to culture the larvae within the adipose tissue (improved motility) together in the same culture well with an extra insert containing the FTSM (improved growth). After setting up the monitoring methods, also direct culture in a FTSM with an integrated adipose tissue could be performed.

To further clarify this aspect, we added the future directions of experiments to the discussion in lines 717 – 723 we discuss the combination of the FTSM and adipose tissue to achieve a synergetic effect of benefits to the larvae: “We hypothesize that by increasing the complexity of the *in vitro* 3-D culturing system will not only achieve a synergetic effect of the FTSM (growth) and the adipose tissue (viability) on larval fitness, but also when combined with direct co-culturing conditions that support monitoring over a prolonged culture period would also potentially enhance further the survival and development of L4 to the pre-adult juvenile worms (as already done) and hopefully also to the more mature adult worms.”

To facilitate monitoring of larvae viability and development, first we need to establish and apply advanced imaging methods to overcome the discussed problems of the direct culture.

This is further discussed in the discussion in line 666 – 672: “To further study whether this interaction is beneficial, we will need to overcome the challenges we had with the direct contact co-cultures. Particularly, advanced microscopy methods need to be established and applied to enable non-invasive monitoring of larval fitness during the long-term cultures versus determining the vitality and growth of larvae using assays that are irreversible such as the digestion of the collagen scaffold followed by MTT (3-(4,5-dimethylthiazol-2-yl)-2,5-diphenyltetrazolium bromide) assay.”

We hope that we were able to address the reviewer’s comment adequately.

We thank the reviewer for the thorough evaluation of the manuscript and the constructive comments.
